# Tumor Associated Macrophages in Kidney Cancer

**DOI:** 10.1155/2016/9307549

**Published:** 2016-10-11

**Authors:** Olga V. Kovaleva, Daria V. Samoilova, Maria S. Shitova, Alexei Gratchev

**Affiliations:** ^1^Institute of Carcinogenesis, NN Blokhin Russian Cancer Research Center, Moscow, Russia; ^2^Medical Faculty Mannheim, Ruprecht-Karls University of Heidelberg, Mannheim, Germany

## Abstract

Tumor associated macrophages (TAMs) are an important element of tumor stroma. They originate from blood monocytes attracted by chemokines and cytokines produced by tumor cells and, being instructed by tumor microenvironment, develop into potent tumor-supporting cell population. TAMs were demonstrated to directly stimulate tumor cell proliferation and to promote angiogenesis. Further TAMs provide for efficient immune escape by producing immunosuppressive cytokines and facilitate tumor dissemination by producing extracellular matrix remodeling enzymes. In renal cell carcinoma (RCC), numerous studies were performed for elucidation of the role of TAM in tumor progression. Using pan-macrophages marker CD68 and type 2 macrophage (M2) markers CD163 and CD206, it was demonstrated that increased density of TAMs is associated with poor survival of patients. Although most of the studies are focused on M2 population in RCC, several markers rather typical for type 1 macrophages (M1) were also characterized. Macrophages isolated from RCC tumors were shown to produce proinflammatory cytokines TNF*α*, IL-1*β*, IL-6, and CCL2. It can be concluded that RCC is an excellent example of a tumor with hybrid phenotype of TAMs that share both M1 and M2 properties. Moreover, TAMs seem to be an attractive therapeutic target as well. Further investigations are needed for identification of RCC-specific TAM markers with high predictive capacity and/or suitable for therapeutic targeting.

## 1. Introduction

Malignant potential of a solid tumor is not only a result of cell transformation, but also a result of a complex interaction of cancer cells with supporting stroma. The latter is composed of fibroblasts, endothelial cells, and inflammatory infiltrate which may be really complex containing various cells of the hematopoietic system especially neutrophils, monocytes, and macrophages. All these cells together generate microenvironment providing better opportunity for tumor growth and invasion. Analysis of stromal cells by various histological techniques or flow cytometry led to accumulation of data supporting the importance of these cells for tumor development. Numerous molecular markers of tumor stromal cells were identified that demonstrate good diagnostic and predictive capacity. Special attention during the past 3 decades was given to tumor associated macrophages (TAMs) since the identification of their alternative ways of activation [1–3]. As other solid tumors, kidney tumors are also comprised of a heterogeneous microenvironment of both malignant and normal stromal cells and contain a large amount of macrophages [4].

Renal cell carcinoma (RCC) is the most frequent form of kidney cancer. It accounts for more than 90% of all kidney cancer cases. Kidney cancer is the 10th frequent malignancy and it is ranked 2nd among cancers with fastest increase of incidence after prostate cancer [5]. The highest incidence of kidney cancer is observed at the age of 70 and it is twice as frequent in male as in female [6]. During the past 2 decades, the incidence of kidney cancer increased worldwide. Kidney tumors show high metastatic potential and are metastatic at diagnosis in about 25% of cases [5]. Prognosis of metastatic kidney cancer is poor. Without specific treatment, the life expectation after metastasis detection is not more than 10–13 months [5]. Risk factors for the development of RCC include cigarette smoking, obesity, continued misuse of pain medications, acquired cystic kidney disease, hypertension, and other genetic diseases [5, 6]. Modern classification of kidney tumors takes into account morphologic, cytogenetic, and molecular properties and defines 5 types of kidney tumors: clear cell renal cell carcinoma (60–85%) and renal papillary carcinoma (7–14%) both arising from epithelial cells of the proximal tubule; and chromophobe renal cell carcinoma (4–10%), benign oncocytoma (2–5%), and collective duct cancer (1-2%) that arise from the intercalating cells of the collecting ducts of the kidney [7].

The major problems in treating RCC are late diagnosis and poor response to available therapies. Therefore, there is an urgent need for new diagnostic and therapeutic approaches reaching beyond the properties of cancer cells.

## 2. Tumor Associated Macrophages Origin and Function

Tumor associated macrophages are considered to be type 2 macrophages (M2) according to widely accepted classification of macrophage activation. Type 2 macrophages were first described in 1992 by Stein et al. as alternatively activated macrophages. In the original work, activation by IL-4 was described as alternative and CD206 (macrophage mannose receptor) was suggested as a marker of this type of activation [8]. Following research led to accumulation of significant amount of data on the markers of M2 and the factors involved in the generation of this cell population. M2 population appeared to be highly heterogeneous from molecular and functional points of view [9, 10]. However, their main functional peculiarities related to the suppression of immune reaction, extracellular matrix remodeling, and angiogenesis stimulation remain to be the most important for their negative contribution to tumor development [3]. Though the concept of M1/M2 dichotomy is being reconsidered now [11], we will use this nomenclature in this review for the sake of clarity and simplicity.

The concept of two types of macrophage activation was developed at the end of the 20th century to complement the Th1/Th2 paradigm [12–14]. M1, also called classically activated macrophages, are characterized by the expression of bactericidal effector molecules and opsonic receptors (Fc*γ*RI, II, and III) [15]. The M1 phenotype develops in response to endogenous inflammatory stimuli such as the Th1 cytokine IFN*γ* or to exogenous inflammatory stimuli such as lipopolysaccharide (LPS) or other bacterial products. M1 stimulate inflammatory reactions by secreting the proinflammatory cytokines. M2, or alternatively activated macrophages, are characterized by the expression of nonopsonic receptors, for example, the macrophage mannose receptor [8] CD163 [16] and hMARCO [17, 18], by upregulation of Th2-associated cytokines and chemokines such as IL-1ra [19, 20] or AMAC-1 [21], and by the production of extracellular matrix components and ECM remodeling factors (fibronectin, tenascin-c, and MMP12) [18]. M2 have markers and functions similar to tumor associated macrophages [22].

Properties of M2 that help in the progression of the tumor include stimulation of angiogenesis that is achieved through the release of angiogenic factors, such as cytokines and matrix metalloproteinases. Extensive angiogenesis requires extracellular matrix degradation, proliferation and migration of capillary endothelial cells, and differentiation of these cells into mature capillaries. Newly formed capillaries provide the tumor with sufficient amounts of nutrients and oxygen and give metastatic cells a route to exit the tumor into circulation [22]. Matrix metalloproteinases (MMPs) play a key role in cell invasion. Matrix metalloproteinases constitute a family comprising more than 20 enzymes that are capable of destroying the protein components of the extracellular matrix [23]. MMPs are important not only for metastasis, but also for normal processes involving extracellular matrix remodeling and normal cell migration, like wound healing or physiological angiogenesis. It should be noted that in the case of tumor invasion stromal cells are the main producers of MMP and tumor cells only stimulate this process by expression of different chemokines, cytokines, and specific inducers. The function of MMPs is not only the degradation of extracellular matrix proteins, but also the activation of some growth and angiogenesis regulators, immobilized in matrix. In particular, it was demonstrated that MMP-9 and MMP-2 take part in the proteolytic activation of growth factor TGF*β*. Similarly, angiogenic (VEGF family members) and antiangiogenic (angiostatin, thrombospondin) factors can be released by metalloproteinases [23]. Almost in all types of tumors overexpression of MMPs correlates with increased invasiveness and poor prognosis. Recent works show that MMPs expression increased in renal tumors and this increase has prognostic value. For example, MMP-2 protein expression is significantly associated with histological grade, TNM stage, tumor size, and LNM in RCC, suggesting that MMP-2 may serve as a biological marker for the prognosis in RCC [24].

ECM remodeling is a complex process that involves different enzymatic systems. Next to MMPs, the system of plasminogen activation is the second most important for tumor pathology. Numerous clinical and experimental studies provided enough evidence to conclude that urokinase-like plasminogen activator (uPA) and its regulators are actively involved in the formation of the metastatic phenotype of many tumor types. uPA is a soluble serine protease, which binds to its membrane receptor uPAR, which leads to activation of the proteolytic activity of uPA. The main function of uPA is the proteolytic cleavage of plasminogen to form plasmin. Plasmin, in turn, has broad substrate specificity and may cleave various ECM proteins including fibronectin, vitronectin, laminin, and fibrin. Similarly to MMPs, plasmin activates growth factors SF/HGF, *β*FGF, and TGF*β* by proteolytic cleavage of their latent forms and activates collagenase by cleavage of procollagenase. All these activities of uPA system contribute to tumor growth and invasion [25]. Increased expression of proteins involved in uPA activation was shown in many types of tumor [26] and increased uPA activity correlates with poor disease prognosis. Experimental models provided additional evidence for the importance of uPA for tumor progression. Inhibition of uPA activity was demonstrated to suppress tumor invasion and metastasis [26]. Expression of uPA and its receptor by TAMs was demonstrated for breast [27] and other types of tumors, where they are involved in the degradation of the ECM which is necessary for the generation of new vessels. The level of uPA receptor expression correlated with the density of blood vessels in tumors and poor prognosis of the disease [28, 29]. In renal cancer, high levels of uPA and uPAR in tumor tissue extracts are associated with a significantly shorter survival of ccRCC patients though without distant metastases [30]. These data indicate the importance of uPA regulated by tumor associated macrophages in the reorganization of the vascular system of the tumor.

## 3. Tumor Associated Macrophages in Kidney Cancer

TAM presence and association of its amount and markers with ccRCC prognosis were demonstrated using histological techniques in 2011 by Komohara et al. [31]. The authors used CD68 as a general macrophage marker and CD163 and CD204 for the identification of macrophage phenotype. They demonstrate that most of CD68+ TAMs in ccRCC also express CD204 which indicates that these are type 2 macrophages. Also CD68+ cells expressed CD163. In the same study, the authors demonstrate that direct coculture of macrophages with RCC cells induces type 2 macrophage phenotype. This is explained by the expression of membrane-type M-CSF on the surface of RCC cells [31]. Another study, performed on TAM population isolated from RCC, showed expression of CD68 and CD163, but not CD206, which may be a result of the different methodology [32]. In the latter study, the authors demonstrated that TAMs isolated from ccRCC produce significant amounts of CCL2—a CC-chemokine that attracts monocytes to tumor site. At the same time, these macrophages produced high amounts of immunosuppressive IL-10. Notably, the authors demonstrated that TAMs from larger tumors produce higher amounts of IL-10 [32]. This finding may indicate that either larger tumors are more potent in generating conditions favorable for M2 programming or stronger immunosuppressive M2 provide for faster tumor growth. In the same study by Daurkin et al., macrophages isolated from ccRCC showed enhanced eicosanoid production via activated 15-lipoxygenase-2 pathway [32], which is typical for activation of macrophages by TGF*β* [10]. Though unexpected, another specific property of TAMs in RCC is expression of CCR8 associated with higher activity of Stat3-mediated signaling, which is rather typical for inflammatory phenotype. These cells are considered to be capable of stimulating FoxP3 expression in T-cells and have proangiogenic activity [33].

Proinflammatory properties of TAMs in RCC were demonstrated on cultures of macrophages isolated from primary tumors. Production of high amounts of IL-6, TNF*α*, and IL-1*β* was demonstrated. In contrast, primary monocyte derived macrophages from the same patients did not produce these cytokines without LPS stimulation. In the same study, TAMs were demonstrated to stimulate proliferation of established RCC cell lines and short-term established RCC cell lines [34]. IL-1*β* is an important factor for tumor angiogenesis and for stimulation of tumor invasiveness. It is capable of inducing matrix metalloproteinases MMP-1, MMP-3, MMP-10, and MT1-MMP in RCC cell lines and stimulates invasiveness of RCC as demonstrated in a mouse model [35]. IL-1R dependent mechanism was shown to be important for the development of protumor macrophage population in a mouse model [36]. Another macrophage-derived proinflammatory cytokine TNF was demonstrated to be important for the induction of cancer stem cell marker CD44 overexpression on ccRCC tumor cells [37]. At the same time, the density of macrophages expressing M2 marker CD163 appears to be more closely related to CD44 expressing cancer cells [37].

There are also markers of TAMs that are not classified to M1 or M2 phenotype. One of these markers is T-cell immunoglobulin and mucin domain-containing molecule-3 (TIM-3). High amounts of TIM-3+ TAMs in ccRCC were associated with poor prognosis of the disease [38].

Accumulated data allow us to conclude that TAMs in RCC show a mixed M1/M2 phenotype. Analysis of the balance between M1 and M2 was performed by Xu et al. on a cohort of 185 RCC patients using histological techniques. The authors selected CD68 as a general macrophage marker, CD11c as a marker of M1, and CD206 as a marker of M2. Statistical analysis revealed that CD68 alone has a poor predictive value, while low CD11+ and high CD206+ as single variables correlated with reduced survival. At the same time, combined analysis of CD11c and CD206 showed the best predictive value. Patients had best survival prognosis if CD11c+ density was high and CD206+ density was low [39]. Interestingly, CD68 was not needed in this analysis. This study is a very good example of the importance of a complex analysis, using markers for both M1 and M2 phenotypes. It would be interesting, however, to see whether there are 2 independent populations of macrophages in RCC or the same cells show a mixed M1/M2 phenotype.

Another highly important feature of TAMs is induction of angiogenesis. In most of the tumors, TAMs are considered to be the source of VEGF which leads to increased microvessel density. RCC is not an exception. In a study performed on a cohort of 51 RCC patients, it was found that high CD68+ TAM density correlates with high microvessel density. Also the levels of VEGF determined by ELISA were found to be higher in RCC compared to normal kidney. The level of VEGF correlated well with diagnostic chance (symptomatic), growth type (interrapid), angiography findings (hypervascular), and tumor size (≥7 cm) [4]. These data are supported by the study, demonstrating that VEGFR1 knockdown leads to a reduced macrophage infiltration in the tumor [40].

**Figure 1:**
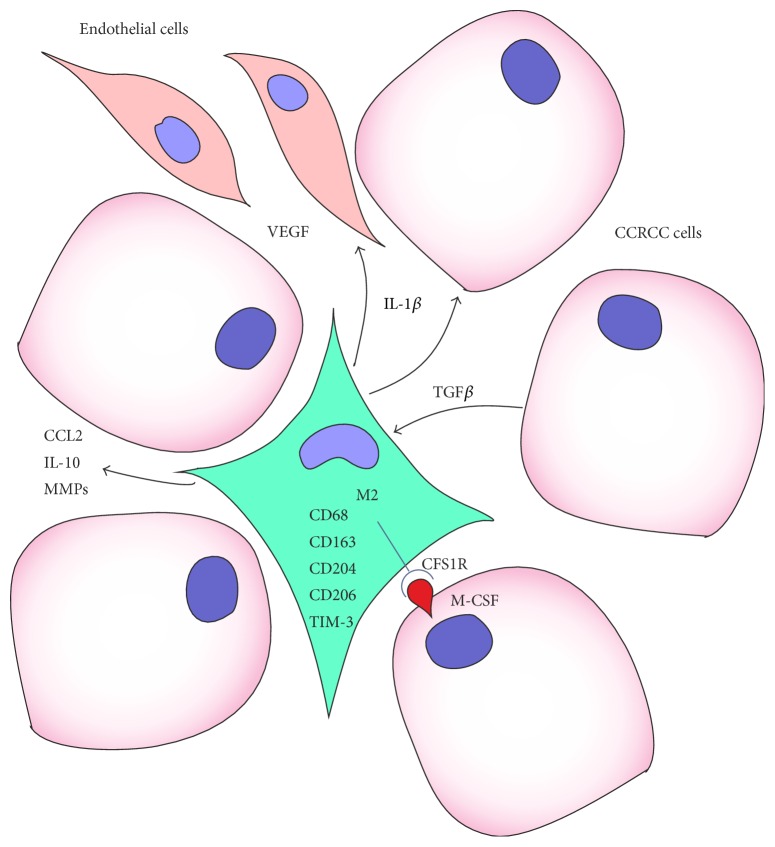
Interaction between tumor cells, tumor associated macrophages, and endothelial cells in renal cell carcinoma.

Taken together, a following model of TAM in RCC can be envisioned (Figure 1). Macrophages infiltrating tumor show a heterogeneous cell population with both M1 and M2 properties. These cells produce CCL2 that attracts new monocytes to support and/or renew the TAM population and inhibitory cytokine IL-10 to downregulate their antitumor activity. RCC cells support monocyte infiltration by producing MMPs and influence their differentiation by membrane bound M-CSF. Proinflammatory cytokine IL-1*β* and VEGF, produced by TAMs, stimulate MMPs production by tumor cells and angiogenesis, respectively.

Although most of the studies are performed on clear cell RCC, the importance of TAMs was demonstrated also for papillary renal cell carcinoma. Papillary renal cell carcinoma (RCC) can be subdivided into subtypes I and II based on histological criteria. Type II is associated with poor prognosis. Although density of CD68+ TAM was similar in both subtypes, the analysis of more specific markers revealed that nearly all macrophages in type II tumors express CD163, while in type I tumors there were less than 30% CD163 positive. Higher number of CD163 cells was associated with higher density of capillaries as defined by CD31 staining. These findings suggest the functional impact of TAMs on the prognosis of papillary RCC type II [41].

## 4. Concluding Remarks

The role of tumor associated macrophages in the pathogenesis of RCC is well established. More research is needed to develop novel and effective diagnostic or therapeutic approaches for RCC. To date, mostly traditional M2 markers (CD206, CD163) or pan-macrophage marker CD68 is used to quantify macrophages in RCC. However, usage of these markers for simple quantification of macrophages is not sufficient for prediction of clinical outcome of the disease or can be even misleading since CD68 was reported to be expressed by ccRCC cells [31]. More TAM markers should be studied, selected from the spectrum of known M2 markers. These can be FXIIIa, fibronectin, *β*IG-H3, Stabilin-1, YKL-39, SI-CLP, tenascin-C, LOX-1, MARCO, and others [9, 18, 42–44]. Use of combinations of these markers can allow the development of a novel diagnostic approach of high predictive capacity. Further, it is highly important to take into account the area of the tumor where TAM analysis is performed. It was described that analysis of macrophages in different parts of the tumor has different predictive value and may express different markers [45]. Current therapeutic targeting strategies for TAMs develop in the similar direction as diagnostic approaches. They utilize markers like CD204, CD206, or folate receptor beta [46] that are not very specific for tumor associated macrophages and even more so not for specific type of tumor. Screening studies have to be performed for identification of tumor specific TAM markers. Results of such screenings will enable development of targeting strategies aimed at reprogramming or elimination of tumor-supporting TAM populations.
